# Branchial cysts: an unusual cause of a mediastinal mass: a case report

**DOI:** 10.1186/s13256-015-0680-y

**Published:** 2015-09-29

**Authors:** Vihar Kotecha, Alex Muturi, Josiah Ruturi

**Affiliations:** 1 Department of Surgery, University of Nairobi, 30197, Nairobi, 00100 Kenya; 2 Department of Cardiothoracic Surgery, Kenyatta National Hospital, Hospital Road, Nairobi, 00202 Kenya

## Abstract

**Introduction:**

Complex embryological processes form the head and neck of humans. It is not flawless; remnants lead to sinuses or cysts, commonly in the head and neck region.

**Case presentation:**

We present the a case of an 8-year-old boy, a primary school pupil, from rural Kenya with chronic cough, wheezing, difficulty in breathing and dyspnea on exertion. He was treated with antibiotics and antitubercular drugs without improvement prior to referral to our hospital. A computed tomography scan of his chest revealed a superior mediastinal mass extending into his neck. A diagnosis of a brachial cleft cyst was made and our patient underwent a successful excision of the mass through a median strenotomy and neck dissection.

**Conclusions:**

Branchial cysts of the neck are common, accounting for 20% of pediatric neck masses. Usually they present as a neck mass but in our case it presented as a mediastinal mass, which is a very rare clinical presentation. Surgical excision is the mainstay of treatment. To the surgeon, the embryology and anatomy should be absolutely clear as dissection may be challenging due to the close proximity and variable course of the cystic stalk to major neck vessels and nerves.

## Introduction

The development of the head and neck is a complex embryologic process. It commences with the formation of branchial clefts and pouches (branchial apparatus) during the fourth week of gestation [1], fusion of the branchial apparatus marks the completion of head and neck development after which the primitive structures formed continue to differentiate and proliferate into the well-defined end products in the head and neck as seen in a normal fetus at birth [1]. Branchial anomalies are remnants of the six main pairs of branchial arches, the associated clefts and pouches that fail to regress or develop abnormally. Anomalies of the second branchial arch are the most common with a prevalence of 90–95% of all branchial cleft cysts [2, 3]. Anomalies of the third and fourth branchial apparatus are the least common with a prevalence of 2–8% [3]. Although branchial apparatus anomalies are a common cause of neck swelling, rarely do they extended into the mediastinum [4]. Clinical presentation is similar to that of other mediastinal masses such as wheezing, dyspnea, cough, chest pain and dysphagia. However, due to the rarity of this diagnosis, it is often not high on the surgeon’s list of differential diagnoses [4]. Radiology may aid in defining the mass but usually a diagnosis is made during surgery where the cysts stalk can be traced back into the neck [4]. The management is often delayed due to its clinical presentation. Surgical excision through a strenotomy with complete excision of the cyst and its neck component is the operation of choice [5]. We present a case of a mediastinal fourth branchial cyst in a pediatric patient from Kenya.

## Case presentation

An 8-year-old African boy, a primary school pupil, from rural Kenya presented to our unit with a 1-year history of cough, difficulty in breathing and dyspnea on exertion that were relieved by rest. His difficulty in breathing was worse at night and there were no reported known relieving factors. His dyspnea was progressive in spite of antibiotics, inhalers and treatment for pulmonary tuberculosis (PTB) for 6 months. His cough was dry and associated with constitutional symptoms; he had no history of hemoptysis, chest pain or foreign body inhalation. Our patient had no features of cardiac failure, no dysphagia or odynophagia. He had been treated for PTB at the district hospital without improvement.

There was no history of similar illness nor was there a history of tumors in the family. Our patient was well prior to the onset of these symptoms with no history of previous hospital admission. A chest X-ray was done after completion of the anti-tubercular medication since there was no improvement. After the X-ray, the patient was referred to our facility. When we examined our patient, he was tachypneic at 40 breaths/minute, with flaring of alae nasi and no cyanosis. He had wheezing with symmetrical chest expansion and his trachea deviated to the left with reduced air entry on the right and rhonchi on auscultation. The results of his cardiovascular and abdominal examinations were unremarkable. His neck had no visible swelling nor was a mass palpable.

**Fig. 1:**
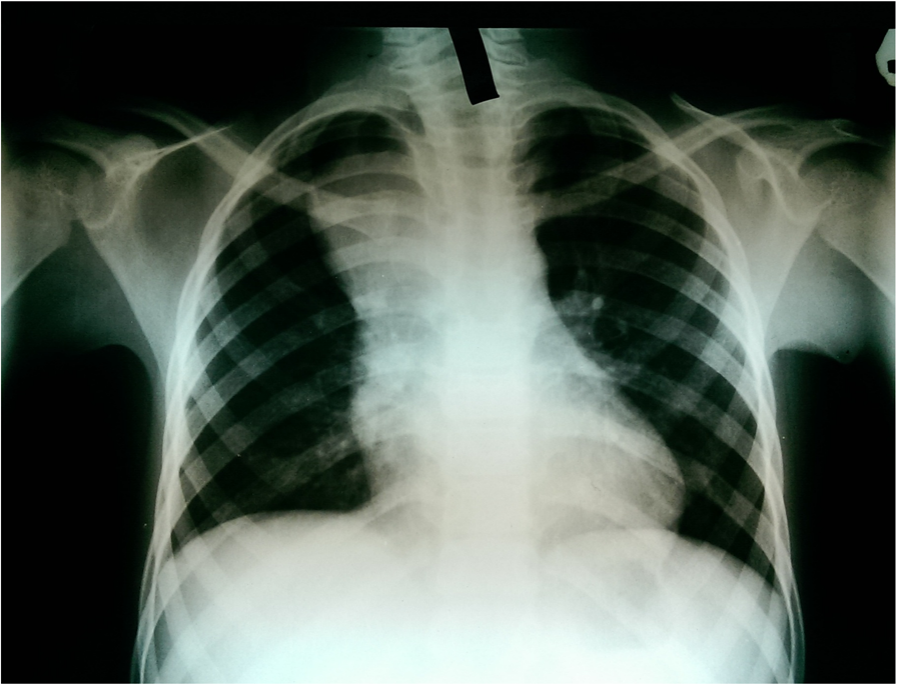
Chest X-ray of the patientᅟ

**Fig. 2:**
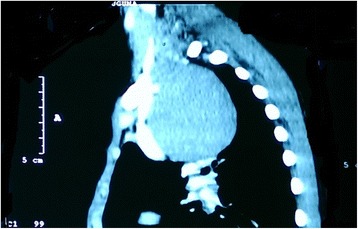
Computed tomography scan of the chest

The chest X-ray done at the district hospital after no improvement on anti-TB medication showed a widened mediastinum without features of PTB (Fig. 1). His hemogram and erythrocyte sedimentation rate were normal. A chest computed tomography (CT) scan was done upon admission to our unit. It showed a cystic mass in the superior mediastinum. It appeared to be arising from the neck at the level of the thyroid cartilage deep to the sternocleidomastoid muscle and extending into the mediastinum to the level of the carina. The mass displaced the major neck vessels (Fig. 2). The lung parenchyma and the heart were reported as normal, with no pleural effusion. The radiologist reported a differential diagnosis of branchial cleft cyst and thymoma.

Our patient was prepared for surgery after the basic preoperative laboratory tests i.e. total blood count, urea electrolytes and creatinine were done and found to be normal. Median sternotomy was performed combined with neck dissection. Intraoperative findings included a right cystic mass arising from the angle of the right jaw extending into the superior mediastinum; there was no extension into the anterior mediastinum. The right common carotid artery was displaced anterolaterally, the superior vena cava anteriorly. The cyst was dissected from the surrounding vessels and the other adjacent structures within its vicinity in the chest. Extension into the neck was noted as the cyst stalk and excised en bloc. The stalk ended abruptly at the angle of the jaw. Its end could not be traced into the pharynx due to the above finding. Postoperatively, our patient was admitted in our intensive care unit from where he was discharged to the general ward after 2 days. He was discharged from the hospital 10 days after the surgery following an uneventful postoperative course. Histopathology of the specimen reported a fibrous cyst wall lined with low columnar and cuboidal cells. These features are consistent with a branchial cyst.

Our patient was seen 2 weeks after discharge in our clinic and all his symptoms had cleared.

## Discussion

Branchial cysts of the neck are a common differential diagnosis of neck masses in the pediatric population. They account for 20% of pediatric neck masses [3, 6]. A differential diagnosis of a branchial cyst as a cause of a superior mediastinal mass is usually forgotten due to its rare occurrence. Our patient did not have a neck mass but the persistent obstructive airway symptoms suggested something more than a cardiopulmonary pathology as the cause of his symptomatology. Mediastinal lesions usually present with dyspnea, cough, chest pain, and dysphagia. Two fifths of such masses in the superior mediastinum are asymptomatic [4]. Despite the courses of antibiotics, including anti-tuberculous medication, no improvement was noted. Given the disparity in history and clinical findings a chest X-ray was requested. X-rays are usually cheap and commonly available as the first line of investigation in the developing world. Our patient had a wide mediastinum on X-ray (Fig. 1). Lack of features of tuberculosis or pneumonia on the X-ray necessitated a chest CT scan to establish the etiology of his symptomatology. A CT scan of his chest assisted in narrowing down a list of differential diagnoses of an already uncommon group of masses [7].

A wide array of investigations for branchial cleft anomalies are available but their use is tailored to the clinical suspicion of the type and origin of the congenital anomaly [3]. For fourth branchial cleft cysts an esophagoscopy, barium swallow, laryngoscopy are investigations of choice to complement the radiologic investigation [3]. Based on the CT scan findings no other investigations were done to complement the diagnosis since cost was a limiting factor. A stepwise approach to investigating this patient was necessary with the investigations mentioned above, but the CT scan gave us adequate answers to proceed with surgery. In our setup, getting all these investigations is limited by cost and the time spent in obtaining them, hence we try to select investigations that will be most beneficial to us in terms of making a diagnosis and deciding the method of treatment. Surgery is the treatment of choice in branchial cleft anomalies due to recurrent infections with these lesions. Irrespective of the type of branchial cleft anomaly, that is a fistula, sinus or a cyst, complete surgical excision is necessary so as to minimize chance of recurrence, which is usually very high following incision and drainage of a third and fourth branchial cyst at 89–94% [3]. To the surgeon, the embryology and anatomy should be absolutely clear as dissection may be challenging due to the close proximity and variable course of the cystic stalk to major neck vessels and nerves [1, 3]. Our patient underwent a successful excision through a median strenotomy and the cyst was followed into his neck and excised completely.

## Conclusions

Neck masses are common among the pediatric age group with branchial cleft anomalies being the cause in a fifth of these patients. Rare occurrence of the third and fourth branchial apparatus anomalies with mediastinal extension requires a thorough clinical evaluation. Anomalies of the fourth branchial cleft in the neck are rare. Their extensions into the chest have only been reported in case reports. Such an anomaly is picked up incidentally while investigating the patient for other conditions; complete surgical excision remains the gold-standard of treatment.

## Consent

Written informed consent was obtained from the patient’s parent for publication of this case report and any accompanying images. A copy of the written consent is available for review by the Editor-in-Chief of this journal. 

## Abbreviations

CT: computed tomography
PTB: pulmonary tuberculosis
